# High Throughput Screening Identifies a Novel Compound Protecting Cardiomyocytes from Doxorubicin-Induced Damage

**DOI:** 10.1155/2015/178513

**Published:** 2015-06-07

**Authors:** Szabolcs Gergely, Csaba Hegedűs, Petra Lakatos, Katalin Kovács, Renáta Gáspár, Tamás Csont, László Virág

**Affiliations:** ^1^Department of Medical Chemistry, Faculty of Medicine, University of Debrecen, Debrecen 4032, Hungary; ^2^Department of Cardiology, Faculty of Medicine, University of Debrecen, Debrecen 4032, Hungary; ^3^MTA-DE Cell Biology and Signaling Research Group of the Hungarian Academy of Sciences, Debrecen 4032, Hungary; ^4^Department of Biochemistry, Faculty of Medicine, University of Szeged, Szeged 6720, Hungary

## Abstract

Antracyclines are effective antitumor agents. One of the most commonly used antracyclines is doxorubicin, which can be successfully used to treat a diverse spectrum of tumors. Application of these drugs is limited by their cardiotoxic effect, which is determined by a lifetime cumulative dose. We set out to identify by high throughput screening cardioprotective compounds protecting cardiomyocytes from doxorubicin-induced injury. Ten thousand compounds of ChemBridge's DIVERSet compound library were screened to identify compounds that can protect H9C2 rat cardiomyocytes against doxorubicin-induced cell death. The most effective compound proved protective in doxorubicin-treated primary rat cardiomyocytes and was further characterized to demonstrate that it significantly decreased doxorubicin-induced apoptotic and necrotic cell death and inhibited doxorubicin-induced activation of JNK MAP kinase without having considerable radical scavenging effect or interfering with the antitumor effect of doxorubicin. In fact the compound identified as 3-[2-(4-ethylphenyl)-2-oxoethyl]-1,2-dimethyl-1H-3,1-benzimidazol-3-ium bromide was toxic to all tumor cell lines tested even without doxorubicine treatment. This benzimidazole compound may lead, through further optimalization, to the development of a drug candidate protecting the heart from doxorubicin-induced injury.

## 1. Introduction

Doxorubicin (DOX) is an anthracycline compound originally isolated from bacteria of the* Streptomyces* genus and used extensively for the treatment of various types of cancer [1–3]. Acute leukemias, Hodgkin and non-Hodgkin lymphomas, osteosarcoma, Ewing sarcoma, breast cancer, neuroblastoma, and small cell lung cancer respond well to DOX monotherapy or combination therapy [4, 5]. Even though doxorubicin and other anthracycline compounds such as daunorubicin have been used by oncologists for more than four decades, their mechanism of action is still not fully understood [6]. Inhibition of topoisomerase II*β*, generation of reactive oxygen species, DNA intercalation and triggering a signaling cascade that involves increased ceramide production, cleavage of the ER membrane protein CREB3L1, nuclear translocation of the N-terminal fragment of this protein, and transcriptional activation of genes that inhibit cell proliferation have been suggested to be responsible for the antitumor effect of anthracyclins [6–8].

The clinical use of DOX is limited mainly by its severe cardiotoxic effect, which may lead to irreversible cardiomyopathy and heart failure [9]. Incidence of heart failure shows close correlation with the cumulative dose of the drug so that it is suggested not to exceed 550 mg/m^2^ [10, 11].

Cardiotoxicity is indicated by morphological alterations (myofibrillar disarray and vacuolization) as observed in cardiac biopsy specimens. Moreover, leakage of troponin can also be detected in the peripheral blood and shows positive correlation with the intensity of heart damage [12, 13]. The mechanism of doxorubicin cardiotoxicity is complex and is closely linked to production of reactive oxygen species. These form indirect electron exchange between the oxygen molecule and the anthracyclines' quinone moiety and can also be produced in redox cycling of doxorubicin-iron complexes [14–16]. Superoxide, hydrogen peroxide, hydroxyl radical, and peroxynitrite have all been implicated in DOX-induced cardiac injury [17, 18]. The role of redox stress is also supported by observations that oxidative stress-induced signaling pathways (e.g., p38 MAP kinases, poly(ADP-ribose) polymerase-1, matrix metalloproteinases, etc.) and metabolic alterations also contribute to the cardiotoxic effects of DOX [19–22]. Moreover, a series of animal experiments has also demonstrated the effectiveness of a ferroporphyrin antioxidant [18], a vitamin E prodrug [23], or a poly(ADP-ribose) polymerase (PARP) inhibitor [24] in preventing or suppressing the cardiotoxic effect of DOX. In recent years topoisomerase 2*α* has emerged as a central mediator of DOX-induced cardiac injury [25]. While topoisomerase 2*β* (expressed mostly in proliferating cells) is considered as the primary target of DOX in tumor cells, topoisomerase 2*α* (expressed by quiescent cells) has been made responsible for suppression of antioxidant enzyme expression, inhibition of mitochondrial biogenesis, and activation of p53 and p53-mediated apoptosis with all of these cellular events implicated in DOX-induced heart failure [25].

Despite our increasing knowledge on the mechanism of DOX-induced heart injury, it still represents an unsolved medical problem necessitating more mechanistic studies as well as the development of novel agents for the prevention of the side effect of anthracyclins. Here we report a screening strategy for the identification of potentially cardioprotective compounds with the capacity to prevent DOX-induced cardiomyocyte injury. With this HTS approach we identified 3-[2-(4-ethylphenyl)-2-oxoethyl]-1,2-dimethyl-1H-3,1-benzimidazol-3-ium bromide (EODB) as a novel compound protecting cardiomyocytes from DOX-induced damage without interfering with its tumor killing activity.

## 2. Materials and Methods

### 2.1. Materials

Dimethyl-sulfoxide, ABTS (A1888), DMEM medium (Gibco 41966), copper(II) chloride dihydrate (307483), neocuproine (N1501), calcein-AM (17783), sulforhodamine B (230162), horseradish peroxidase (P8375), xanthine (X4002), xanthine oxidase (X4500), nitroblue tetrazolium (NBT) (N6876), superoxide dismutase (S7571), and Ampliflu Red (90101) were purchased from Sigma-Aldrich (Saint Louis, MO, USA). RPMI 1640 cell culture medium (BE12-115F), glutamine (BE17-605F), and fetal bovine serum (DE14-802F) were purchased from Lonza (Basel, Switzerland). DIVERset 10 000 compound library was purchased from ChemBridge (San Diego, CA, USA). Doxorubicin was purchased from Teva (Debrecen, Hungary).

### 2.2. Cell Culture

#### 2.2.1. Cell Lines

H9C2 cells were cultured in DMEM (10% FBS and 2 mM glutamine, 5 g/L glucose). A549, Jurkat, and THP-1 cell lines were cultured in RPMI 1640 medium supplemented with 10% FBS and 2 mM glutamine. SAOS-2 cell line was cultured in DMEM (10% FBS and 2 mM glutamine, 1 g/L glucose).

#### 2.2.2. Primary Neonatal Rat Cardiomyocyte Culture

Primary neonatal cardiomyocyte culture was prepared from 1–3-day-old Wistar rats as described earlier [26, 27]. Pups were killed by cervical dislocation, and then the hearts were harvested and rinsed in ice-cold PBS buffer. The ventricles were then chopped and digested in 0.25% trypsin for 25 min. To increase the number of cardiomyoblasts in the cell suspension, 90 min preplating was applied in 10% FBS-containing DMEM. Then cells were plated at 1.5 × 10^4^ cell/well density in 96-well plates with 10% FBS-containing DMEM supplemented with 1% glutamine and antibiotic/antimycotic solution. Cells were maintained in a humidified incubator (37°C, 5% CO_2_). After 24 hours, the medium was changed to DMEM containing 1% FBS to help cardiomyoblast differentiation.

### 2.3. MTT Viability Assay

For the HTS screening H9C2 cells (7 × 10^3^/well) were plated to 96-well plates one day before the treatment. Compounds of the library were transferred to the plates with a Tecan Freedom EVO liquid handling robot (100 nL/well) to reach 10 *μ*M final concentration. Each compound was tested on two separate microplates. (Controls received the corresponding concentration of the vehicle DMSO). After 30 min incubation at 37°C, cells were treated with doxorubicin (final concentration: 300 ng/mL) or cell culture medium (control). Samples were incubated for 24 hours at 37°C. 10 *μ*L MTT solution (5 mg/mL) was added to the samples (final concentration: 0.5 mg/mL). Samples were incubated for 120 min, at 37°C. Supernatants were aspirated and replaced by DMSO (50 *μ*L/well). Measurement was performed with a Thermo Multiskan reader at 540 nm. Viability data were expressed as the percentage of control. Compounds with more than 20% efficiency were regarded as cardioprotective.

The non-HTS (manual) version of the assay was carried out the same way except for the fact that test compounds were given to the cells with manual pipettes. The assay protocol differed for nonadherent cell lines (Jurkat and THP-1). In this case, cells (10^5^/well) were seeded into 96-well plates and were treated with EODB and DOX as described above. 24 h after DOX treatment, cells were transferred to V-bottom plates. MTT was added, and cells were incubated as described above. Plates were then spun down (1600 rpm, 5 min), medium was aspirated, and DMSO (75 *μ*L) was added to dissolve the cells and the formazan crystals. Fifty microliter aliquots were transferred to flat bottom plates for determination of absorbance as described above.

### 2.4. Cytotoxicity Assay Based on Cell-Covered Area

H9C2 cells (7 × 10^3^/well) were seeded into 96-well plates one day before the treatment. Cells were treated with the hit compounds of the screening, in the final concentration of 12 *μ*M. (Controls received the corresponding concentration of the vehicle DMSO). After 30 min incubation at 37°C, cells were treated with doxorubicin (300 ng/mL final concentration) or cell culture medium (control). Samples were incubated for 24 hours at 37°C. Cell culture medium was changed to Coomassie staining solution (0.1%) and incubated for 20 minutes. Samples were washed once with PBS and dried. From each condition altogether nine images were taken (3 wells from 3 independent experiments) with a Leica SP8 microscope and were used for the calculation of cell-covered area in each condition. Image analysis performed with Tscratch software was used to determine the size of cell-covered areas.

### 2.5. Calcein-Based Viability Assay

Cell viability of H9C2 cells (7 × 10^3^/well) and two-day-old primary rat neonatal cardiomyocytes (1.5 × 10^4^ cell/well) was also determined with the calcein-AM assay. Cells were pretreated with 12 *μ*M (for H9C2 cells) or 5–25 *μ*M (for primary cells) of compound#10 or its vehicle (DMSO) for 30 minutes followed by 300 ng/mL doxorubicin treatment for 24 hr. Compound#10 treatments were maintained during the doxorubicin challenge. At the end of the protocol, viability was determined by calcein-AM assay [28]. After washing twice with D-PBS, primary cardiomyocytes were incubated with 1 *μ*M calcein AM (Sigma, Saint Louis, MO) for 30 min at room temperature. In living cells, the cell-permeable calcein AM (nonfluorescent) is hydrolyzed by intracellular esterases to calcein (green-fluorescent). Fluorescence intensity was measured following a washing step by means of a fluorescence plate reader (Fluostar Optima, BMG Labtech, Ortenberg, Germany) using 490-nm excitation and 520-nm emission filters.

### 2.6. Visualization of Cellular Morphology

For the concentration finding experiment and for illustrating alterations in the morphology and number of cells following DOX treatment, we stained cell cultures (H9C2 in concentration finding experiments; A549 and SAOS-2 cells in the antitumor experiments) with the Coomassie dye as described under Section 2.4.

### 2.7. Caspase-3 Activity Measurement

Measurement of caspase activity (from both H9C2 (3 × 10^4^/well) and primary cardiomyocyte cultures (5 × 10^4^ well)) was performed as described before [29].

### 2.8. Combined Measurement of Viability and Necrotic Cell Death

Survival and necrotic death of H9C2 cells (7 × 10^3^/well) has been determined by sequential measurement of the activity of lactate dehydrogenase (LDH) released by the cells into the culture supernatants (as described before [30]) followed by determination of cell viability using the calcein-AM assay as described above.

### 2.9. Measurement of Cell Proliferation

Cell proliferation was determined with the sulforhodamine B (SRB) assay [31]. The assay is based on the ability of the protein dye sulforhodamine B to bind electrostatically and in a pH dependent manner to basic amino acid residues of proteins in trichloroacetic acid- (TCA-) fixed cells. Under mild acidic conditions SRB binds to the cells and under mild basic conditions it can be extracted and solubilized for measurement. Cells were seeded into 24-well plates at a density of 2.5 × 10^3^ and treated with 12.5 *μ*M EODB and 300 nM DOX alone or in combination for 5 days. (Controls received the same amount of DMSO vehicle as the EODB samples.) Cells were then fixed with 10% TCA for 1 hour at 4°C, washed 5 times with distilled water and air dried. Cells were stained with 0.4% (w/v) sulforhodamine B (SRB) solution in 1% acetic acid for 10 minutes. Unbound dye was removed by washing 5 times with 1% acetic acid and plates were air dried. Bound stain was solubilised with 10 mM Tris base and absorbance was read at 540 nm using a Multiskan MS plate reader (Labsystems, Vantaa, Finland). Proliferation capacity is expressed as percentage of vehicle control.

### 2.10. Measurement of Free Radical Scavenging with the ABTS Decolorization Assay

Measurement of the radical scavenging activity of EODB was performed as described before [32] with slight modifications as follows. ABTS decolorization assay utilizes a chromogenic free radical, the radical monocation of 2,2′-azinobis-(3-ethylbenzothiazoline-6-sulfonic acid) (ABTS^•+^), which is decolorized during reduction by hydrophilic or lipophilic antioxidants. ABTS^•+^ radical cation was generated by oxidation of ABTS with potassium persulfate overnight one day before the experiment. ABTS was dissolved in water to a 7 mM concentration and this stock solution was allowed to react with potassium persulfate (2.45 mM final concentration) followed by incubation in the dark at room temperature. Before the experiment, absorbance of the ABTS^•+^ solution was adjusted to 1.2 at 405 nm. Test compounds were incubated with the ABTS^•+^ solution for 30 minutes at RT. Assays were done in 96-well plates in triplicate. Absorbance was measured with Victor V^3^ multilabel reader (405 nm). Antioxidant activity was expressed as the percentage of control (DMSO-treated) samples and was compared to the effect of 12 *μ*M Trolox.

### 2.11. Cupric Ion Reducing Antioxidant Capacity (CUPRAC) Assay

Measurement of the antioxidant capacity of EODB was performed as described by Apak et al. [33], with the following modifications: 12 *μ*M of Trolox was used as positive control, final volume was reduced to 100 *μ*L, and the measurement was performed in 96-well microplates.

### 2.12. H_2_O_2_ Scavenging Activity

Hydrogen-peroxide scavenging capacity was measured using the Ampliflu Red reagent (10-acetyl-3,7-dihydroxyphenoxazine) in a cell-free system. 12 *μ*M EODB or 10 *μ*M ascorbic acid was incubated with 0.1 *μ*M H_2_O_2_ (Sigma-Aldrich), 50 *μ*M Ampliflu Red reagent, and 0.1 U/mL horseradish peroxidase in phosphate buffered saline for 5 minutes at room temperature. H_2_O_2_, in the presence of horseradish peroxidase, reacts stoichiometrically with Ampliflu Red reagent to generate the red-fluorescent oxidation product, resorufin. Fluorescence was read with excitation at 530 nm and emission at 590 nm using a Fluoroskan Ascent FL plate reader (Labsystems, Vantaa, Finland).

### 2.13. Superoxide Scavenging Activity

Superoxide scavenging capacity was measured with the NBT (nitroblue tetrazolium chloride) reduction test as described [34] with modifications as follows. Superoxide was produced by the xanthine/xanthine oxidase system and NBT reagent (50 *μ*M final concentration) was used to detect superoxide. NBT is reduced to the blue NBT diformazan product by superoxide [34]. 12 *μ*M EODB or 100 *μ*M ascorbic acid (positive control) was incubated with xanthine oxidase (0.1 U/mL) and xanthine (50 *μ*M), in potassium phosphate buffered saline (0.067 M, pH7.8, supplemented with 0.7 mg/mL BSA to keep the produced NBT diformazan in solution and with 0.5 mM EDTA to chelate transition metals) in 200 *μ*L final volume for 2 minutes at room temperature. As a vehicle control, DMSO was applied in the concentration present in the EODB samples. The xanthine/xanthine oxidase reaction was also run in the presence of 500 U/mL superoxide dismutase (SOD) and SOD-inhibitable NBT reduction was considered to be due to superoxide production. Absorbance of NBT diformazan was measured at 540 nm using a Multiskan MS plate reader (Labsystems, Vantaa, Finland).

### 2.14. Western Blotting

Cells (3 × 10^6^/sample) were washed once in PBS and collected by scraping into 200 *μ*L of ice-cold lysis buffer (62.5 mM Tris-HCl, pH 6.8, 2% SDS, 10% glycerol, 50 mM DTT, 1 mM PMSF, 1 mM NaF, 1 mM Na_3_VO_4_, and protease inhibitors). The extracts were further lysed with sonication, and the supernatant was collected after centrifugation. Protein concentrations were determined with the BCA reagent (Thermo Scientific). Proteins (30 *μ*g/well) were separated in 10% SDS-PAGE and transferred to nitrocellulose membranes. Membranes were blocked with 5% BSA in Tris-buffered saline (TBS) for 1 hour. Primary antibodies against JNK/stress-activated protein kinase, phospho-JNK/stress-activated protein kinase (Thr183/Tyr185) (cell signaling technology), were applied overnight at 4°C. After three washes in TBS containing 0.05% Tween 20, secondary antibodies (peroxidase-conjugated goat anti-rabbit IgG, cell signaling technology) were applied for 1 h. Blots were washed three times in TBS containing 0.05% Tween 20 and once in TBS, incubated in enhanced chemiluminescence reagent (SuperSignal Chemiluminescent Substrate, Pierce). Bands were evaluated by densitometry using ImageJ software.

### 2.15. Statistical Analysis

All experiments were performed three times on different days. Analysis of variance was performed by one way ANOVA followed by Tukey's test for statistical analysis and for the determination of significance with *P* < 0.05 considered as significant.

## 3. Results and Discussion

### 3.1. Results

#### 3.1.1. Screening for Cardioprotective Compounds Protecting from Doxorubicin Toxicity

**Figure 1:**
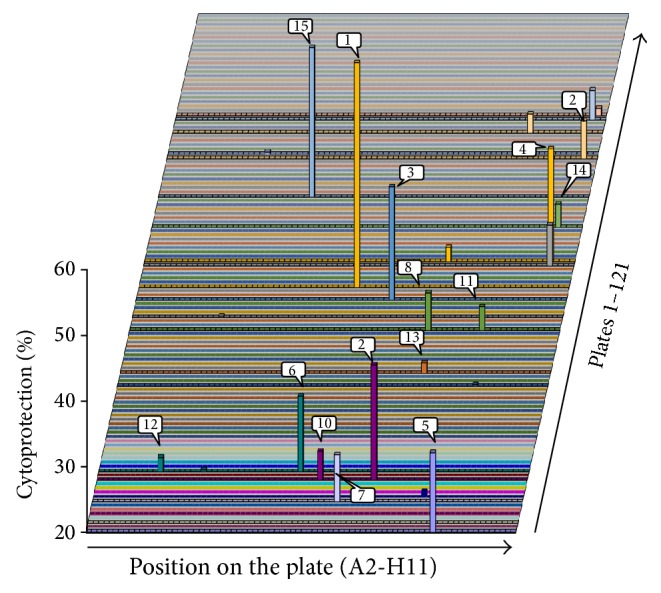
Screening for cardioprotective compounds. H9C2 cells were treated with test compounds (10 *μ*M) for 30 min followed by a 24 h exposure to DOX (300 ng/mL). Viability was determined with the MTT assay. Percent cytoprotection is plotted so that only the most effective 15 compounds showing higher than 20% of cytoprotective effects appear as “hits.” (SD values are not shown due to the 3D presentation of data).

**Figure 2:**
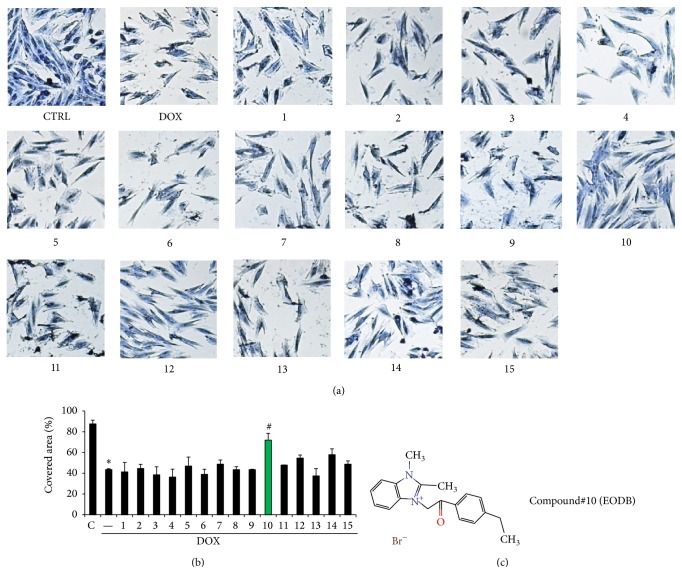
Reassessment of cardiocytoprotective effect of hit compounds with a microscopy-based method. H9C2 cells were treated with the hit compounds (12 *μ*M) for 30 min followed by a 24 h exposure to DOX (300 ng/mL). Cells were stained with Coomassie dye and photographed with a Leica MC120 HD camera connected to a Leica DM IL LED microscope (5x magnification) (a). Cell-covered area was determined with the Tscratch software (b). Mean ± SEM of 3 independent experiments was calculated (b). Only compound#10 had significant (^#^ *p* < 0.05) cytoprotective effect in this assay. The structural formula of compound#10 (EODB) is shown on panel (c).

We have screened the ChemBridge DIVERset compound library consisting of 9680 compounds. For this we used H9C2 rat cardiomyocytes and determined cell viability 24 h after doxorubicin treatment (Figure 1). Compounds showing at least 20% cardioprotection were considered potentially cardioprotective. Fifteen compounds met these criteria and were used in subsequent experiments. According to our experience a drawback of MTT-based or similar dehydrogenase activity-based viability assays is the frequent occurrence of false positive hits. Therefore we have analyzed the morphology of cells after DOX treatment and retested the 15 primary hit compounds (Figures 2(a) and 2(b)). By determining the surface area occupied by living cells we have detected decreased viability in DOX-treated samples. Out of the 15 compounds retested only compound#10 appeared to convincingly exert protective effect in DOX-treated H9C2 cells (Figures 2(a) and 2(b)). This compound is 3-[2-(4-ethylphenyl)-2-oxoethyl]-1,2-dimethyl-1H-3,1-benzimidazol-3-ium bromide (EODB) (Figure 2(c)).

#### 3.1.2. EODB Protects H9C2 Cells Both from Apoptotic and from Necrotic Cell Death

**Figure 3:**
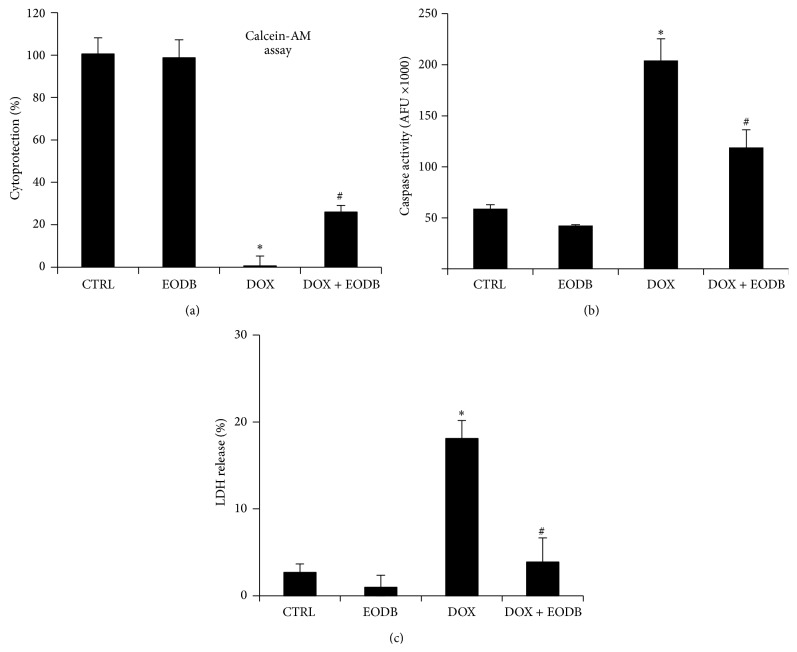
Characterization of the mode of DOX-induced cell death. H9C2 cells were pretreated (30 min) with compound#10 and then treated with DOX (300 ng/mL). After 24 h viability, apoptotic and necrotic cell deaths have been assessed with calcein-AM assay (a), caspase activity (b), and lactate dehydrogenase (LDH) release (c), respectively. The lead compound significantly reduced DOX-induced cytotoxicity with a stronger effect on necrosis than on the apoptotic cell death. Mean ± SEM of 3 independent experiments is presented. Stars indicate significant (^*∗*^ *p* < 0.05) DOX-induced cell death compared to control whereas hatch marks indicate significant (^#^ *p* < 0.05) cytoprotection by the lead compound.

We have further characterized the cytoprotective effect of EODB. First we aimed to find the optimal EODB concentration for the follow-up experiments. Therefore we pretreated H9C2 cultures with different concentrations of EODB and then treated the cells with DOX. Cell cultures were stained with Coomassie dye and visual evaluation suggested 12 *μ*M to be sufficient to provide maximal protection while at higher concentrations (especially at 100 *μ*M) toxicity could be observed (Figure S1 in Supplementary Materials available online at http://dx.doi.org/10.1155/2015/178513). Thus we chose 12 *μ*M for the follow-up experiments. Next we have confirmed the cytoprotective effect of EODB in another assay based on calcein-AM hydrolysis which confirmed its significant cytoprotective effect against DOX-induced toxicity (Figure 3(a)). Doxorubicin-induced cell death has both apoptotic and necrotic features [35, 36]. We have determined cellular caspases-3 activity and release of LDL to assess apoptotic and necrotic cell death, respectively (Figures 3(b) and 3(c)). Pretreatment of the cells with EODB inhibited both caspase-3 activation and LDH release indicating protection from both apoptotic and necrotic cell death.

#### 3.1.3. EODB Protects Primary Rat Cardiomyocytes from DOX-Induced Damage

**Figure 4:**
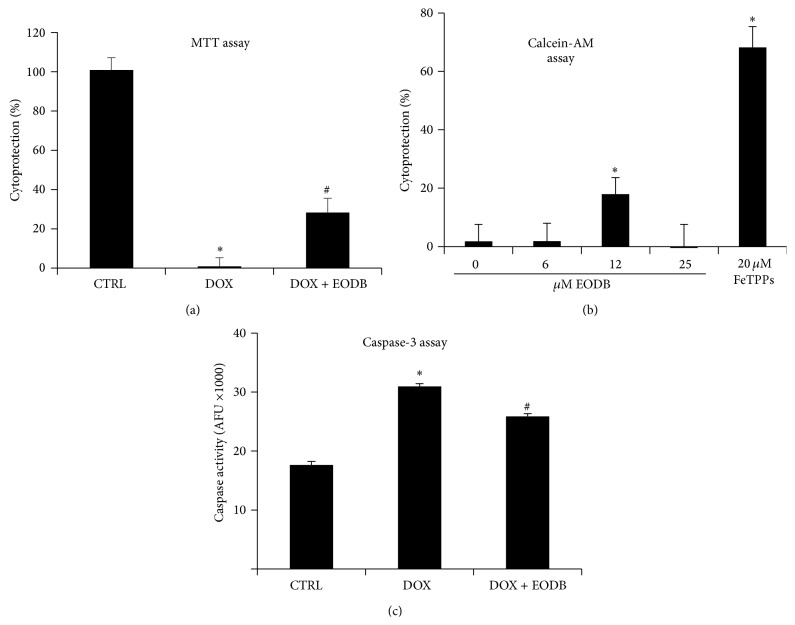
EODB protects primary rat cardiomyocytes from DOX-induced injury. Rat primary cardiomyocytes were pretreated for 30 min with 12 *μ*M EODB or 20 *μ*M FeTPPs (positive control) followed by a 24 h exposure to DOX (300 ng/mL). Viability was assessed with MTT assay (a) and calcein-AM assay (b) and data were expressed as percent cytoprotection. Caspase activity was determined as a marker of apoptosis (c). Mean ± SEM of 3 independent experiments is presented. EODB provided significant (^*∗*^ *p* < 0,05) protection as compared to vehicle in all three assays.

Cell-based screening programs typically utilize immortalized cell lines due to relatively cheap culture, easy manipulation (e.g., gene silencing), and availability of high number of cells. However, the spontaneous or induced mutations that were required for immortalization may alter the biological behavior and responses of these cells. Therefore we have also investigated the effect of EODB on primary rat cardiomyocytes. We found that at 12 *μ*M concentration EODB provided a significant protection from DOX-induced toxicity both in MTT assay (Figure 4(a)) and in the calcein-AM assay (Figure 4(b)). Furthermore, EODB also exerted a significant inhibitory effect on DOX-induced caspase-3 activation (Figure 4(c)). Calcein assay data (Figure 4(b)) also indicated some inherent toxicity of EODB because at 25 *μ*M concentration the cytoprotective effect of the compound vanished.

#### 3.1.4. EODB Lacks Antioxidant Activity but Inhibits JNK Activation

**Figure 5:**
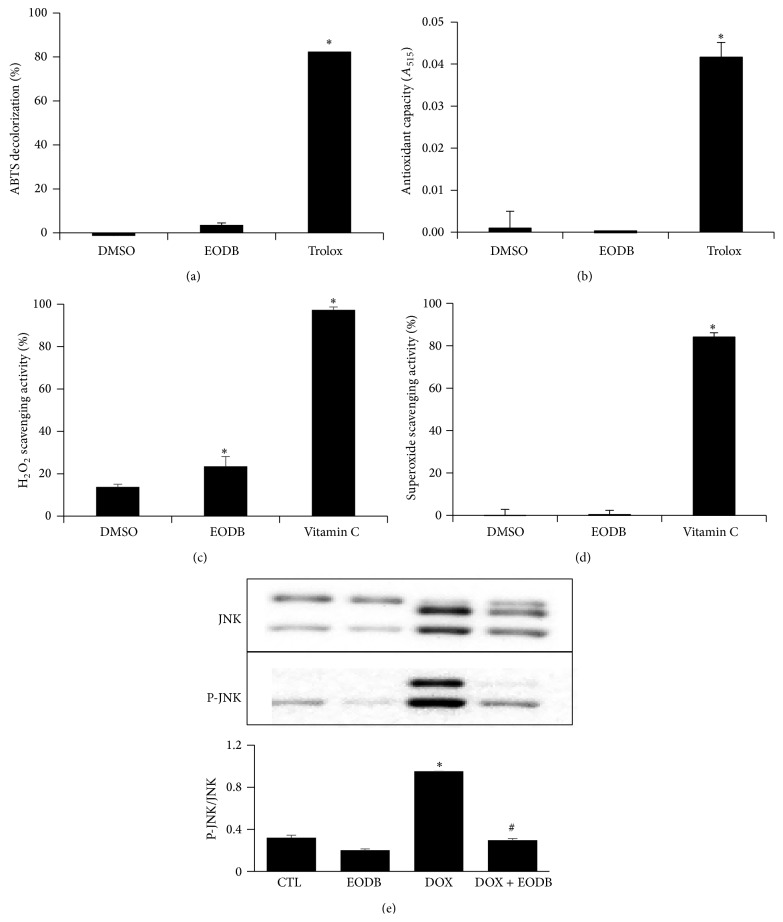
Lack of marked antioxidant effect but inhibition of DOX-induced JNK activation by EODB. We tested the radical scavenging and antioxidant effects of EODB in ABTS assay (a), in the CUPRAC assay (b), in Ampliflu Red oxidation assay (c) and in superoxide assay (d). In the concentration used in the experiments (12 *μ*M) EODB lacked any detectable radical scavenging or antioxidant effect in the ABTS (a), CUPRAC (b) and superoxide (d) assays and displayed a small but statistically significant H_2_O_2_ scavenging activity (c). EODB also lacked superoxide scavenging activity (d). Trolox (12 *μ*M) (a, b) and vitamin C (10 *μ*M on panel (c) and 100 *μ*M on panel (d)) were used as positive controls. Mean ± SEM of three independent experiments is presented. DOX-induced JNK activation (e) has been determined by Western blotting 24 h after DOX treatment (carried out as in the cytotoxicity experiments). (^*∗*^ *p* < 0.05).

Since generation of ROS and RNS is considered as an important event in DOX-induced cardiac damage [37] and many experimental compounds providing protection against DOX-induced cardiotoxicity possess antioxidant effect [38] we set out to determine whether or not EODB can scavenge radicals. First we tested the compound in parallel with Trolox (positive control) in ABTS decolorization assay (Figure 5(a)) but it had no radical scavenging effect. In the CUPRAC assay which detects reducing activity, the compound also showed no such effect (Figure 5(b)). Moreover, we have also investigated whether EODB may neutralize H_2_O_2_ or superoxide. While the positive control vitamin C efficiently inhibited H_2_O_2_-induced oxidation of the fluorescent target molecule and also scavenged superoxide, EODB only had a marginal (although statistically significant) inhibitory effect on H_2_O_2_ (Figure 5(c)) while it did not scavenge superoxide (Figure 5(d)). Thus it appears that the cytoprotective effect of EODB is not likely to be due to its antioxidant effect.

We have also investigated the potential role of two prodeath MAP kinases p38 and JNK in the cytoprotective effect of EODB. While p38 did not seem to be involved in the mechanism (data not shown) JNK was activated in DOX-treated H9C2 cells and EODB suppressed DOX-induced JNK activation (Figure 5(d)).

#### 3.1.5. EODB Does Not Interfere with the Antitumor Effect of Doxorubicin

**Figure 6:**
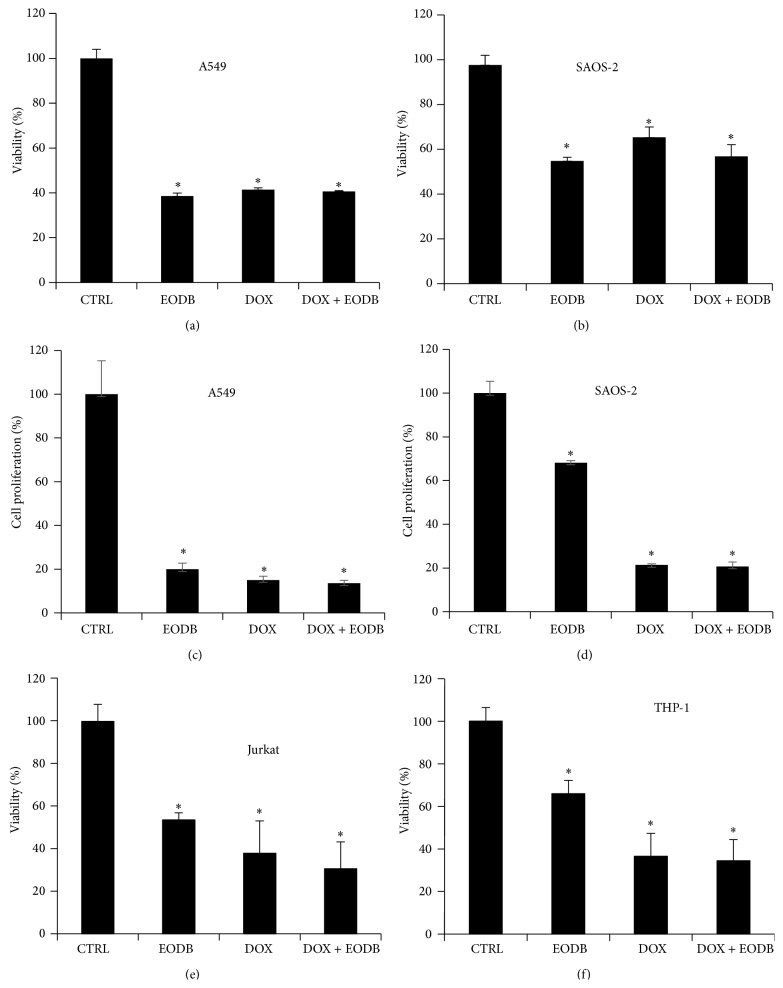
EODB does not interfere with the antitumor effect of DOX. A549 (a, c), SAOS-2 (b, d), Jurkat (e) and THP-1 (f) cells were pretreated with EODB (12 *μ*M) for 30 min and were then incubated with DOX for 24 h (a, b, e, and f) or for 5 days (c and d). Viability (a, b, e and f) and proliferation (c, d) were determined with MTT assay and sulforhodamine B assay, respectively. Mean ± SEM of 3 independent experiments is presented. (^*∗*^ *p* < 0.05).

For a potential drug candidate to be used to protect cardiomyocytes in DOX-treated cancer patients, it is important not to compromise the antitumor effect of DOX. DOX is used in the treatment of different kinds of tumors including cancers of lung [39] and bone origin [40] as well as in different forms of leukemias [41]. Therefore we tested whether or not EODB affects the cytotoxic effect of DOX in A549 lung epithelial carcinoma cells, SAOS-2 osteosarcoma cells, and in monocytic and T cell leukemia cell lines (THP-1 and Jurkat, resp.). We found that EODB did not interfere with the tumor cell killing activity of doxorubicin (Figure 6). Interestingly, EODB alone (without DOX) was toxic to all these cancer cell lines (Figures 6(a), 6(b), 6(e), and 6(f)), an effect we have not observed in H9C2 cell cultures. EODB also strongly inhibited the proliferation of A549 cells whereas its antiproliferative effect on SAOS-2 cells was less marked (Figures 6(c) and 6(d)). Analysis of Coomassie-stained A549 and SAOS-2 cultures also confirmed the toxic effect of EODB on these tumor cell lines (Figure S2).

### 3.2. Discussion

HTS screens are typically applied on potential pharmacological targets implicated in the pathomechanism of diseases affecting large populations. Accordingly, to our best of knowledge, HTS has not yet been used to identify molecules protecting cardiomyocytes from DOX. In our current study we aimed at proving the viability of the HTS approach for the development of pharmacological agents protecting cardiac cells from DOX-induced toxicity. To this end we screened a small but diverse compound library for cardioprotective effect and our viability-based screen identified several hit compounds. However, keeping in mind that methodological issues often render it difficult to draw objective conclusions from cytoprotective assays especially in the case of DOX-induced cardiotoxicity [42], the hit compounds were retested in a different assay based on morphological criteria (measurement of cell-covered surface area). Only one of the test compounds (EODB) passed this double test and in subsequent experiments we characterized the protective effect of EODB. The most likely reason for the discrepancy between the primary screening and the retesting is that most hit compounds probably perturbed the MTT assay leading to false positive results. EODB also protected primary cardiomyocytes from DOX-induced cell death suggesting that our findings may translate well to preclinically more relevant conditions.

So far most experimental compounds that sufficiently protected the heart or cardiomyocytes from DOX-toxicity targeted reactive oxygen or nitrogen species. For example, a vitamin E prodrug [23], flavonoids [43, 44], a peroxynitrite decomposition catalyst ferroporphyrin compound [18], and many other antioxidants proved effective in providing protection from DOX-induced cardiac injury [45]. EODB, however, is not likely to neutralize ROS or RNS species directly. It did not scavenge ABTS or superoxide radicals nor did it test positively in the CUPRAC antioxidant assay and its H_2_O_2_ scavenging activity was not very prominent either. Thus it is quite likely that the cardiocytoprotective effect of EODB is indirect and may interfere with damage-signaling pathways. In the DOX-induced cardiotoxicity model, the lack of antioxidant effect is not incompatible with cardioprotection as indicated by the protective effect of inhibitors of poly(ADP-ribosyl)ation [20], topoisomerase [46], or angiotensin type 1 receptor [47]. EODB may also target a step in one of the many cell death pathways. Our data demonstrated that it inhibits both apoptotic and necrotic cell deaths suggesting that it more likely interferes with a proximal event of damage-signaling rather than specifically targeting one particular cell death pathway. In fact such “indirect” effects may bear higher clinical relevance than direct radical scavenging, because, despite promising preclinical data, antioxidant approaches (e.g., N-acetyl cysteine or iron chelation) were not effective in humans [48, 49]. Cell death promoting MAP kinases may represent ideal targets for cytoprotective approaches [50–52]. Under our assay conditions JNK but not p38 kinase was activated in DOX-treated cells and EODB efficiently inhibited DOX-induced JNK phosphorylation. Whether this represents a direct effect of the compound on the kinase or it inhibits an upstream event in the signaling cascade requires further investigation.

Structural analysis of EODB may also give hints to explain both the protective effect of the compound and its side effects. EODB contains a benzimidazole moiety which may be linked to some of these effects. Benzimidazole derivatives represent a pharmacologically active family of agents with demonstrated antiviral [53, 54], antimicrobial [55, 56], and antidiabetic effects [57]. Furthermore, telmisartan, an angiotensin II receptor blocker, contains two benzimidazole moieties. Interestingly, in a rat model, telmisartan has been shown to limit the cardiotoxic effect of DOX as verified by improved hemodynamic status, suppressed expression of matrix metalloproteinase activity p22(phox), p47(phox), p67(phox), nuclear factor kappa B, and Nox4, and reduced oxidative DNA damage, lipid peroxidation, and cell death [47, 58]. It is tempting to speculate that EODB may also possess angiotensin II receptor blocking effect which might be the key factor underlying its cardiocytoprotective effect. Moreover, compounds with a benzimidazole scaffold also have demonstrated antitumor effect via inhibition of topoisomerases [59–61]. On the one hand this may be important for explaining both the DOX-protective effect (see above) and the toxic effect of EODB on tumor cell lines. EODB did not interfere with the antitumor effect of DOX in the four tumor cell lines tested. In fact, it also proved cytotoxic in the absence of DOX. We have not observed toxicity on H9C2 cells (only at 100 *μ*M) but in primary rat cardiomyocytes the toxicity may have contributed to the limited though significant protective effect of EODB.

In summary, our experiments proved the viability of the cell-based HTS approach for the identification of DOX protective compounds. EODB protected both H9C2 cells and rat primary cardiomyocytes from DOX-induced toxicity without hampering the antitumor effect of DOX. Through structure optimization EODB may serve as a template for the development of compounds protecting heart cells from DOX-induced toxicity. Further investigations are needed to identify the exact molecular target of this promising drug candidate.

## Supplementary Material

Supplementary figure 1: In order to determine the most optimal cytoprotective concentration of EODB, H9C2 cells were pretreated with different concentrations of EODB for 30 min followed by exposure to DOX (300 ng/mL). After 24h incubation, cells were washed and stained with Coomassie dye as described in the "Materials and methods" section. We concluded that 12 μM is the most optimal EODB concentration for further experiments
Supplementary figure 2: In order to visualize the effect of EODB on the morphology of tumor cell lines, A549 and SAOS-2 cells were pretreated with different concentrations of EODB for 30 min followed by exposure to DOX (300 ng/mL). After 24h incubation, cells were washed and stained with Coomassie dye as described in the "Materials and methods" section. We found that EODB sensitized both A549 and SAOS-2 cells to DOX-induced cell death and was toxic to these cells even in the absence of DOX.
