# Supplementary material for: High Throughput Screening Identifies a Novel Compound Protecting Cardiomyocytes from Doxorubicin-Induced Damage
Source: Oxid Med Cell Longev. 2015 Jun 7;2015:178513. doi: 10.1155/2015/178513 (PMC4475553; doi:10.1155/2015/178513)
Supplement: Supplementary file 1 — Supplementary figure 1: In order to determine the most optimal cytoprotective concentration of EODB, H9C2 cells were pretreated with different concentrations of EODB for 30 min followed by exposure to DOX (300 ng/mL). After 24h incubation, cells were washed and stained with Coomassie dye as described in the "Materials and methods" section. We concluded that 12 μM is the most optimal EODB concentration for further experiments Supplementary figure 2: In order to visualize the effect of EODB on the morphology of tumor cell lines, A549 and SAOS-2 cells were pretreated with different concentrations of EODB for 30 min followed by exposure to DOX (300 ng/mL). After 24h incubation, cells were washed and stained with Coomassie dye as described in the "Materials and methods" section. We found that EODB sensitized both A549 and SAOS-2 cells to DOX-induced cell death and was toxic to these cells even in the absence of DOX. [file 178513.f1.pptx]

## Slide 1
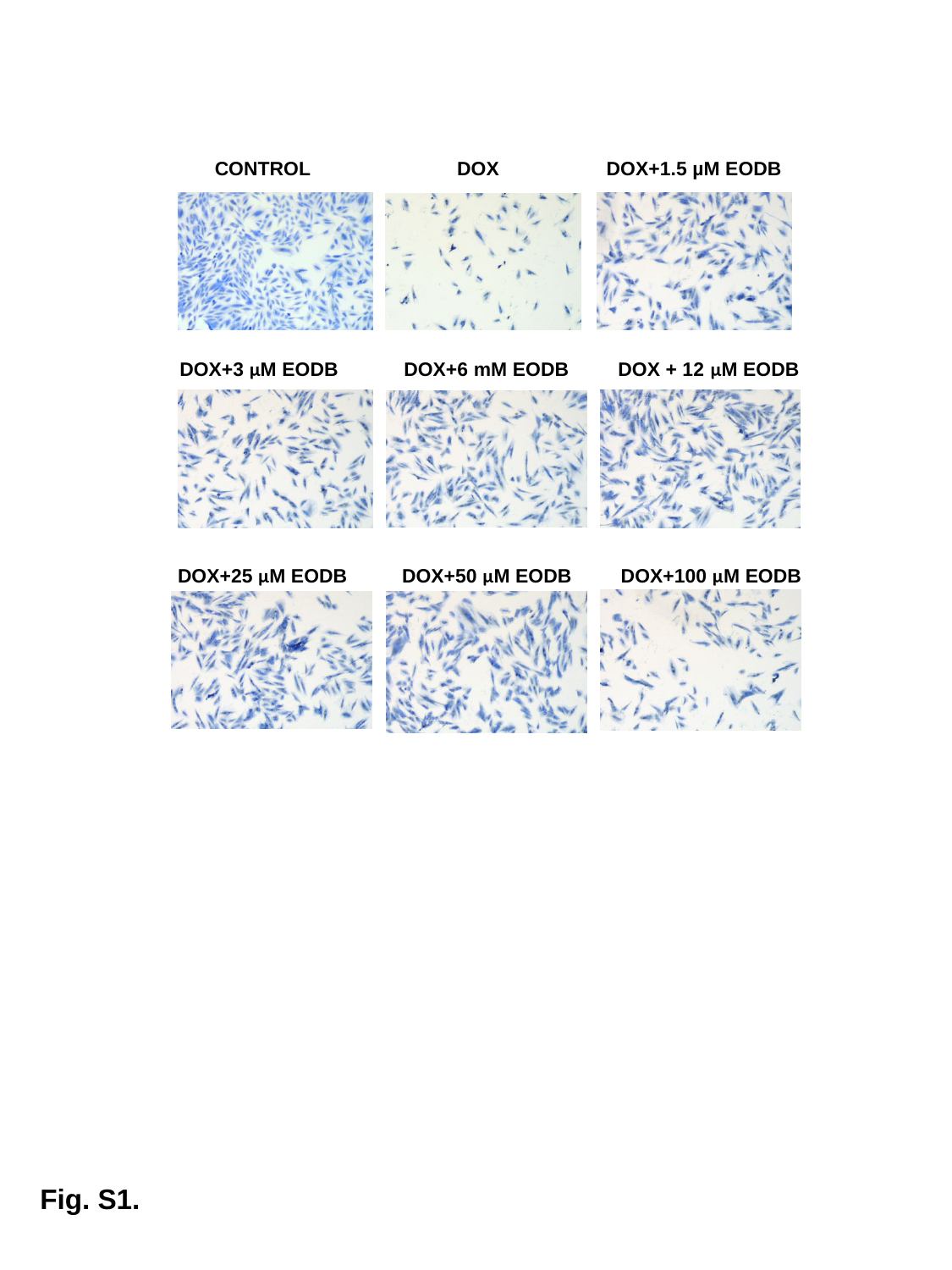

CONTROL
DOX+1.5 µM EODB
DOX
 DOX+3 µM EODB DOX+6 mM EODB DOX + 12 µM EODB
DOX+25 µM EODB DOX+50 µM EODB DOX+100 µM EODB
Fig. S1.
