# Supplementary figures and images for: High Throughput Screening Identifies a Novel Compound Protecting Cardiomyocytes from Doxorubicin-Induced Damage
Source: Oxid Med Cell Longev. 2015 Jun 7;2015:178513. doi: 10.1155/2015/178513 (PMC4475553; doi:10.1155/2015/178513)

## Slide 1
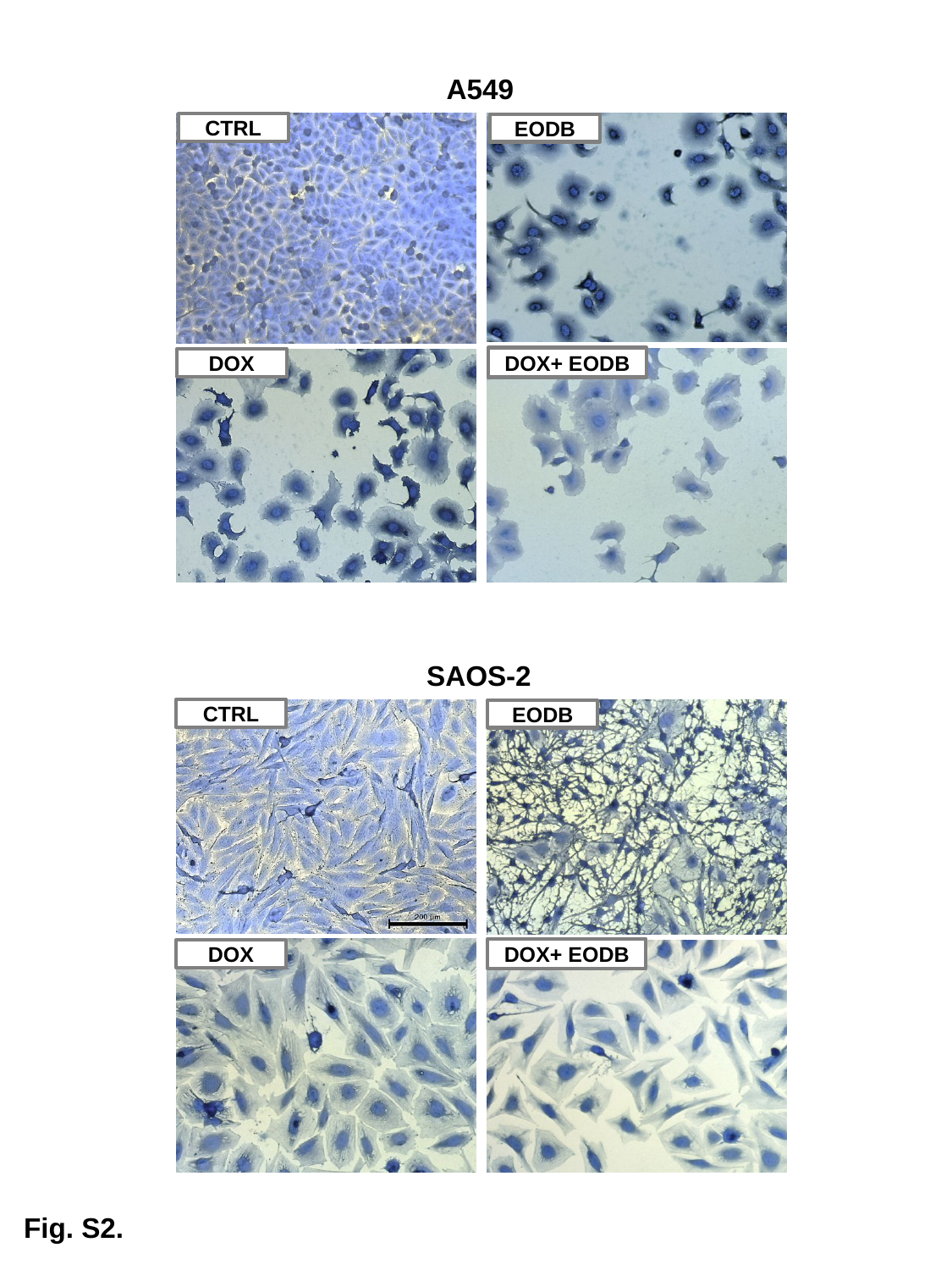

A549
CTRL
EODB
DOX+ EODB
DOX
SAOS-2
CTRL
EODB
DOX+ EODB
DOX
Fig. S2.

Supplement: Supplementary file 2 [file 178513.f2.pptx]
